# Staphylococcus lugdunensis Endophthalmitis Following Intravitreal Anti-vascular Endothelial Growth Factor Injections

**DOI:** 10.7759/cureus.30439

**Published:** 2022-10-18

**Authors:** Umar Ahmed, Lava Nozad, Manuel Saldana-Velez

**Affiliations:** 1 Ophthalmology, East Sussex Healthcare NHS Trust, Eastbourne, GBR

**Keywords:** pars plana vitrectomy (ppv), anti-vegf treatment, staphylococcus lugdunensis, intravitreal injections, acute endophthalmitis

## Abstract

*Staphylococcus lugdunensis* is a rare causative organism of endophthalmitis following intravitreal injections. It presents an aggressive disease course with potentially devastating outcomes. In this case, the patient presented late with a severely painful, red eye with a reduced visual acuity from 6/18 to light perception following bilateral intravitreal injections of anti-vascular endothelial growth factor. Strict adherence to the bilateral intravitreal injection protocol meant prevention of infection in the right eye. Intravitreal vancomycin was administered without delay and an emergency vitreous biopsy was performed, confirming *S. lugdunensis* as the causative organism. An intense course of oral and topical steroids was chosen due to the aggressiveness of this organism. Early vitreo-retinal opinion was sought but the patient was deemed not suitable for vitrectomy due to initial improvements in visual acuity to hand movements. The patient showed improvements in the visual acuity to 1/60, and remains on a weaning regime of oral and topical steroids with no further complications.

## Introduction

Endophthalmitis is a rare but serious complication of intraocular procedures [1]. It can lead to serious implications for patients; it has been reported that 36.1% of cases suffer long-term complications such as non-clearing vitreous debris, retinal detachments, macular oedema, and epiretinal membranes and 31.2% of patients have poor vision of counting fingers or worse even after treatment [2].

Endophthalmitis may be exogenous or endogenous in origin. Exogenous is by far the most common, and within this category, the most common cause for endophthalmitis is ocular surgery, with an incidence of 0.03%-0.6% following cataract surgery [3]. The reported incidence following intravitreal anti-vascular endothelial growth factor (VEGF) infections is 0.028%-0.035% [4]. Endogenous endophthalmitis originates from elsewhere in the body and occurs by haematogenous spread; this is rare and makes up an estimated 2%-8% of all endophthalmitis cases [5].

*Staphylococcus lugdunensis* is a coagulase-negative staphylococcus that is a typical constituent of normal skin flora. It is known to cause severe infections such as native valve endocarditis and osteomyelitis. It is also a rare yet recognised causative organism of acute endophthalmitis, which can follow a more aggressive course of disease than other coagulase-negative organisms, when caused by intravitreal injections [6]. This further highlights the importance of understanding *S. lugdunensis*-associated acute endophthalmitis, including its prevention and management.

## Case presentation

An elderly female presented to the ophthalmology clinic with a three-day history of pain, redness and reduced vision in her left eye. Five days prior to presentation, she had received bilateral intravitreal injections with Eylea (2mg/0.05ml aflibercept) for wet age-related macular degeneration (AMD). This was the most recent of multiple intravitreal injections she had received for wet AMD in both eyes. Her previous ocular history included left and right eye cataract surgery. Her past medical history included hypertension and hypothyroidism.

Routine procedures had been followed for the administration of her bilateral intravitreal injections. Local anaesthesia was achieved with topical proxymetacaine 0.5% (preservative-free), and topical 5% iodine was administered into the eye prior to skin preparation with the povidone-iodine 10% solution. An InVitria device (FCI Ophthalmics, Pembroke, MA) was used to administer the intravitreal injection 4.0mm from the limbus at the infero-temporal position [7]. Topical chloramphenicol drops were administered into the eye immediately following the injection.

Assessment and investigations

Slit-lamp examination of the left eye revealed extensive conjunctival hyperaemia, a hazy and oedematous cornea with a 1.2mm hypopyon and 4+ cells in the anterior chamber. Fundal view was challenging due to the degree of corneal oedema and the presence of a cataract. The visual acuity was light perception only (pre-injection best-corrected visual acuity 6/18). Goldmann applanation tonometry demonstrated an intraocular pressure of 17mmHg. B-scan ocular ultrasound demonstrated vitreous debris and intact appearances of the retina. The right eye demonstrated no acute findings.

**Figure 1:**
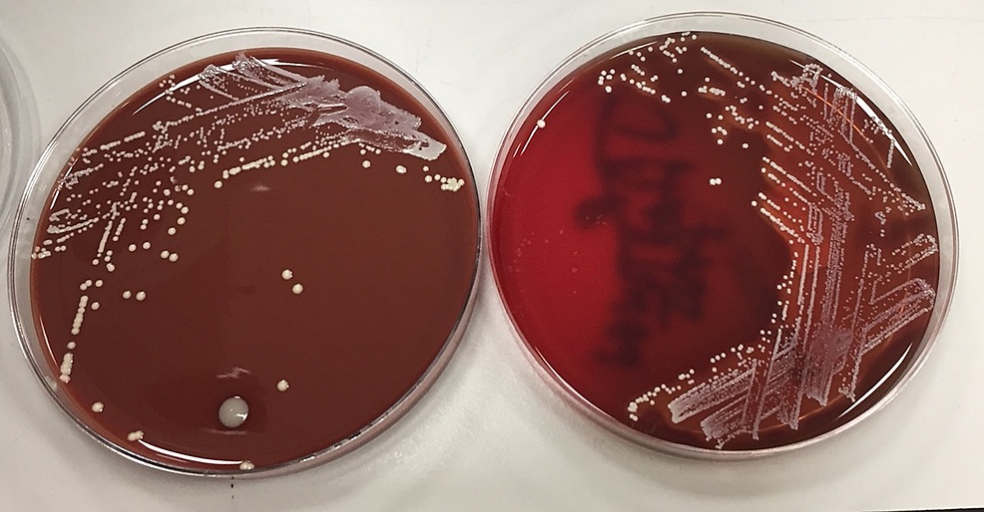
The vitreous sample demonstrating a heavy growth of Gram-positive cocci

**Figure 2:**
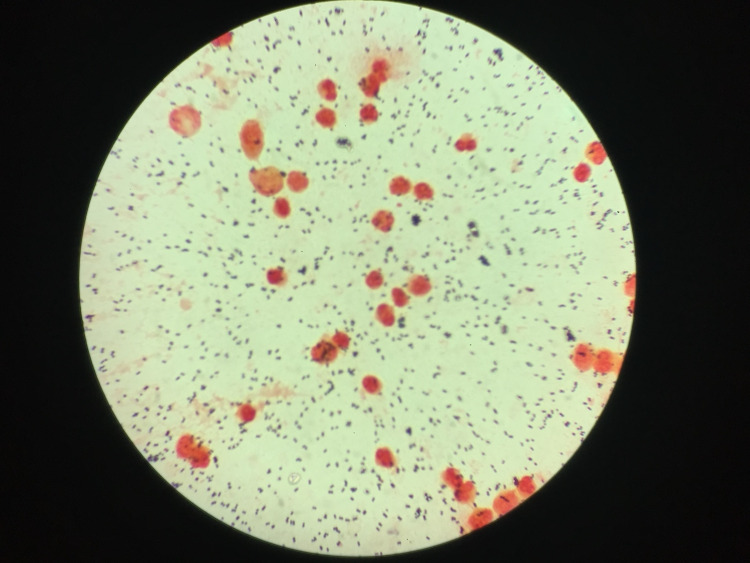
The vitreous sample demonstrating numerous white cells and isolated coagulase-negative Staphylococcus lugdunensis

The patient underwent an emergency vitreous biopsy and injection of intravitreal antibiotics. The vitreous sample demonstrated a heavy growth of Gram-positive cocci and numerous white cells and isolated coagulase-negative *S. lugdunensis*, illustrated in Figure 1 and Figure 2.

Treatment

A single dose of intravitreal vancomycin (0.1ml, 1mg/0.1ml) and ceftazidime (0.1ml, 2mg/0.1ml) was administered and the patient was commenced on a treatment regime as illustrated in Table 1.

**Table 1:** Initial treatment regime for the patient

Initial treatment
Ciprofloxacin oral tablets, 750mg twice daily
Maxidex (dexamethasone 0.1%, 1mg/ml) eye drops, one drop in the left eye four times daily
Cyclopentolate 1% eye drops, one drop in the left eye twice daily
Ciprofloxacin 0.3% eye drops, one drop in the left eye every 2 hours

Early specialist input was sought from the vitreo-retinal surgeons who opted for not performing vitrectomy as there were mild improvements in the clinical condition; her visual acuity had improved to hand movements. The patient also reported less pain in the eye. Hence, instead, she was admitted for intensive topical steroids (preservative-free dexamethasone 0.1%, 1mg/ml eye drops, every two hours) and an additional 1mg dose of intravitreal vancomycin. The patient was discharged with a modified treatment regime as illustrated in Table 2.

**Table 2:** Modified treatment regime for the patient

Modified treatment
Moxifloxacin oral tablets, 400mg once daily
Dexamethasone 0.1%, 1mg/ml eye drops (preservative-free) one drop in the left eye every 2 hours
Chloramphenicol eye drops, one drop in the left eye four times a day
Cyclopentolate 0.5% eye drops, one drop in the left eye twice daily

A further review, nine days following diagnosis, demonstrated persistently low vision and ongoing inflammation in the anterior chamber. The patient was commenced on oral prednisolone (once daily at 1mg/kg body weight) with gastro-protection.

Outcome and follow-up

The patient is currently on a weaning regime of oral and topical steroids. Six weeks following the acute presentation, the eye was found comfortable with a visual acuity of 1/60. On examination, vitreous debris was found; there were no signs of ongoing inflammation otherwise.

## Discussion

It is important to note the lack of randomised controlled trials on the management of acute endophthalmitis following intravitreal injections. Furthermore, there are very few case studies on *S. lugdunensis* as a causative organism. General management principles, however, remain the same as they do for acute post-operative endophthalmitis.

*S. lugdunensis* has the potential to cause aggressive soft tissue infections, in particular, an aggressive manifestation of infective endocarditis [8]. Endophthalmitis caused by *S. lugdunensis *however has only been described in a very few cases, with its nature differing depending on the type of procedure causing it. In one study, it was found that when endophthalmitis was caused by intraocular surgery, it had an insidious presentation with good visual outcomes after treatment [9]. However, when caused by intravitreal injections, there was a more acute and aggressive course with poor visual outcomes after treatment. The poor visual outcomes in patients who experienced post-intravitreal injection endophthalmitis were hypothesised to be due to pre-existing advanced ocular diseases; hence, baseline vision was already poor [6]. Nevertheless, due to *S. lugdunensis* potentially causing an aggressive form of endophthalmitis, early diagnosis is essential for good outcomes, with a prompt anterior chamber tap and vitreous biopsy and intravitreal injections of broad-spectrum antibiotics including vancomycin (0.1ml, 1mg/0.1ml) and ceftazidime (0.1ml, 2.25mg/0.1ml) before biopsy results are returned in order to prevent delay in treatment in accordance with the protocol [10].

A common practice in the management of suspected endophthalmitis involves the injection of intravitreal vancomycin and amikacin or ceftazidime. Vancomycin provides the Gram-positive cover, which is sufficient in treating *S. lugdunensis* infection in most cases. Although *S. lugdunensis* remains sensitive to several antimicrobial agents, there has been some reported variation in resistance worldwide particularly against penicillin G and oxacillin [11].

In the early stages of management, it is crucial to consider pars plana vitrectomy in patients with a visual acuity of light perception or lower. This is in accordance with a landmark trial, the Endophthalmitis Vitrectomy Study, which demonstrated improved visual outcomes in such patients following vitrectomy [12]. It is crucial to note, however, that this trial is now over 20 years old and only includes patients with endophthalmitis following cataract surgery or secondary intraocular lens implantation. Care must be taken when applying such data to other types of endophthalmitis. Furthermore, modern advancements in surgical techniques, in particular, relating to vitrectomy, mean that surgery should be considered in a broader range of patients, i.e. those presenting with a less severe visual loss [13]. The patient in our case was reviewed by the vitreo-retinal surgeons who decided not to proceed with vitrectomy as she had demonstrated improving visual acuity and symptomatic relief following the initial intravitreal antibiotic course.

A case series of patients with *S. lugdunensis *endophthalmitis reported higher numbers of post-vitrectomy retinal detachment compared to other coagulase-negative staphylococci with a rate of 60% compared to 3%, respectively [14]. This was hypothesised to be due to the virulence of this organism causing retinal necroses. The virulence of this organism and its effect on the retina is important to consider when contemplating the administration of further intravitreal antibiotics since these may also be associated with retinal complications. In particular, intravitreal vancomycin has been reported to cause a rare yet devastating condition known as haemorrhagic occlusive retinal vasculitis [15,16].

The overall role of steroids in the management of endophthalmitis has been controversial. Topical steroids are initiated in almost all cases where fungal infection is thought to be unlikely. Trials have demonstrated no substantial benefit with the addition of intravitreal corticosteroids, whereas others have been inconclusive and even demonstrated improved outcomes in patients infected by more virulent organisms [17,18]. Oral steroids, however, are known to have beneficial effects and often make up a part of the ongoing systemic treatment in these patients with severe disease or those who respond poorly to initial management. Since *S. lugdunensis *endophthalmitis has a more aggressive presentation, the use of oral steroid treatment would be appropriate in this case; however, in view of the patient's poor visual acuity of 1/60, it is unlikely to have been of benefit.

The added nuance in this case is that it was a bilateral, same-day intravitreal procedure that led to a unilateral complication with *S. lugdunensis* endophthalmitis. In the literature, there is retrospective data suggesting that bilateral same-day intravitreal procedures are considered safe or even recommended, with some studies suggesting the reduced number of visits may lower the rate of culture-proven endophthalmitis [19,20]. The two intravitreal injections must be treated as two separate sterile procedures, and this exact protocol was followed in this case.

## Conclusions

Post-intravitreal injection endophthalmitis caused by *Staphylococcus lugdunensis* follows an acute and aggressive course of disease, highlighting the importance of prompt recognition and management. Due to this and in the view of the poor outcome for this patient (visual acuity 1/60), *S. lugdunensis* endophthalmitis should be treated more aggressively. In keeping with this outcome, vitrectomy should be undertaken in these patients even though there is an increased risk of post-vitrectomy retinal detachment with *S. lugdunensis*. Additionally, immediate vitrectomy should be considered at the time of vitreous biopsy. Oral steroids are unlikely to be of benefit; they are also more likely to cause complications due to the co-morbidities that are prevalent in these types of patients. Finally, conducting same-day bilateral intravitreal injections does not mean complications of endophthalmitis will occur in both eyes when they are treated as two separate and sterile procedures.
